# CaCO_3_ Precipitation in Multilayered Cyanobacterial Mats: Clues to Explain the Alternation of Micrite and Sparite Layers in Calcareous Stromatolites

**DOI:** 10.3390/life5010744

**Published:** 2015-03-09

**Authors:** Józef Kaźmierczak, Tom Fenchel, Michael Kühl, Stephan Kempe, Barbara Kremer, Bożena Łącka, Krzysztof Małkowski

**Affiliations:** 1 Institute of Paleobiology, Polish Academy of Sciences, Twarda 51/55, 00-818 Warsaw, Poland; E-Mails: kremer@twarda.pan.pl (B.K.); malk@twarda.pan.pl (K.M.); 2 Marine Biological Laboratory, University of Copenhagen, Strandpromenaden 5, 3000 Helsingør, Denmark; E-Mails: tfenchel@bio.ku.dk (T.F.); mkuhl@bio.ku.dk (M.K.); 3 Institute of Applied Geosciences, Technische Universität Darmstadt, Schnittspahnstr. 9, 64287 Darmstadt, Germany; E-Mail: kempe@geo.tu-darmstadt.de; 4 Institute of Geological Sciences, Polish Academy of Sciences, Twarda 51/55, 00-818 Warsaw, Poland; E-Mail: bhlacka@gmail.com

**Keywords:** cyanobacterial mats, CaCO_3_ precipitation, CaCO_3_ morphs, micrite/sparite alternation, δ^13^C in microbial mats, purple sulfur bacteria

## Abstract

Marine cyanobacterial mats were cultured on coastal sediments (Nivå Bay, Øresund, Denmark) for over three years in a closed system. Carbonate particles formed in two different modes in the mat: (i) through precipitation of submicrometer-sized grains of Mg calcite within the mucilage near the base of living cyanobacterial layers, and (ii) through precipitation of a variety of mixed Mg calcite/aragonite morphs in layers of degraded cyanobacteria dominated by purple sulfur bacteria. The δ^13^C values were about 2‰ heavier in carbonates from the living cyanobacterial zones as compared to those generated in the purple bacterial zones. Saturation indices calculated with respect to calcite, aragonite, and dolomite inside the mats showed extremely high values across the mat profile. Such high values were caused by high pH and high carbonate alkalinity generated within the mats in conjunction with increased concentrations of calcium and magnesium that were presumably stored in sheaths and extracellular polymer substances (EPS) of the living cyanobacteria and liberated during their post-mortem degradation. The generated CaCO_3_ morphs were highly similar to morphs reported from heterotrophic bacterial cultures, and from bacterially decomposed cyanobacterial biomass emplaced in Ca-rich media. They are also similar to CaCO_3_ morphs precipitated from purely inorganic solutions. No metabolically (enzymatically) controlled formation of particular CaCO3 morphs by heterotrophic bacteria was observed in the studied mats. The apparent alternation of *in vivo* and post-mortem generated calcareous layers in the studied cyanobacterial mats may explain the alternation of fine-grained (micritic) and coarse-grained (sparitic) laminae observed in modern and fossil calcareous cyanobacterial microbialites as the result of a probably similar multilayered mat organization.

## 1. Introduction

The structure and ecophysiology of modern *calcifying* cyanobacterial mats from various natural settings has become the subject of detailed field and laboratory studies over the past three decades (e.g., [1,2,3,4,5,6,7,8,9,10,11,12,13,14,15,16,17,18,19,20,21,22,23,24,25,26,27,28,29,30,31,32]). Such research is of great importance for paleontologists and biosedimentologists studying cyanobacterial microbialites from the remote geological past. Another incentive is that the results of such studies may permit conclusions to be drawn concerning the character and evolution of early life and its habitats. Such insights are particularly important for the reconstruction of Archean life, which is represented almost entirely by macroscopic biosedimentary structures known as stromatolites, or, more generally, microbialites [33,34,35,36]. These are believed to be products of *in vivo* and/or post-mortem mineralized (mostly calcified) benthic mats of cyanobacteria or cyanobacteria-like microbiota [23,37,38,39].

Ecophysiological observations of modern cyanobacterial mats, combined with mineralogical, geochemical, and taphonomical analyses of fossil cyanobacterial microbialites, enable the reconstruction of some fundamental paleoenvironmental parameters such as bathymetry, illumination, redox potential, pH range, metal stress and eutrophication level. Shallow-water calcareous cyanobacterial microbialites from the late Archean found in South Africa [40] and deep-water siliceous cyanobacterial microbialites from the early Silurian in southern Poland [41,42], are good examples of dramatically different paleoenvironments occupied by ancient cyanobacterial mats.

Although several attempts have been made to study cyanobacterial mats with associated microbiota either *in situ* or with samples transferred to the laboratory (e.g., [16,17,28,30,43,44,45]), extrapolation of these results to Precambrian microbialites should be done with caution. An alternative to these approaches was invented by Fenchel [46], who induced microbial mat growth on top of natural coastal sediment after removal of meio- and macrofauna. The results of >3 years of incubation were published in a series of papers recounting the structure, development and function of the cultured mats [46,47,48,49,50]. In the present paper, we discuss and summarize results of these experiments focusing on the microbiota and processes producing the alternation of fine-grained (**micritic**—crystals less than 4 μm) and coarse-grained (**sparitic**—grains over 4 μm) calcareous laminae characteristic for many *in situ* calcified stromatolites, along with a review of recent studies dealing with the origin of lamination in subfossils and fossil calcareous cyanobacterial microbialites, and with the variety of calcium carbonate morphs precipitated during accretion of these microbialites. To achieve this, we aim herewith: (i) to document carbonate particles (morphs) generated within the cultured cyanobacterial mats and to evaluate factors controlling the origin of these particles, (ii) to elucidate the origin of micritic and sparitic laminae in stromatolites and circumstances responsible for their formation. Both purposes appear to be crucial for understanding the calcification processes recorded in many modern, subfossil, and ancient marine cyanobacteria-dominated microbialites.

## 2. Material and Methods

### 2.1. Samples

**Figure 1:**
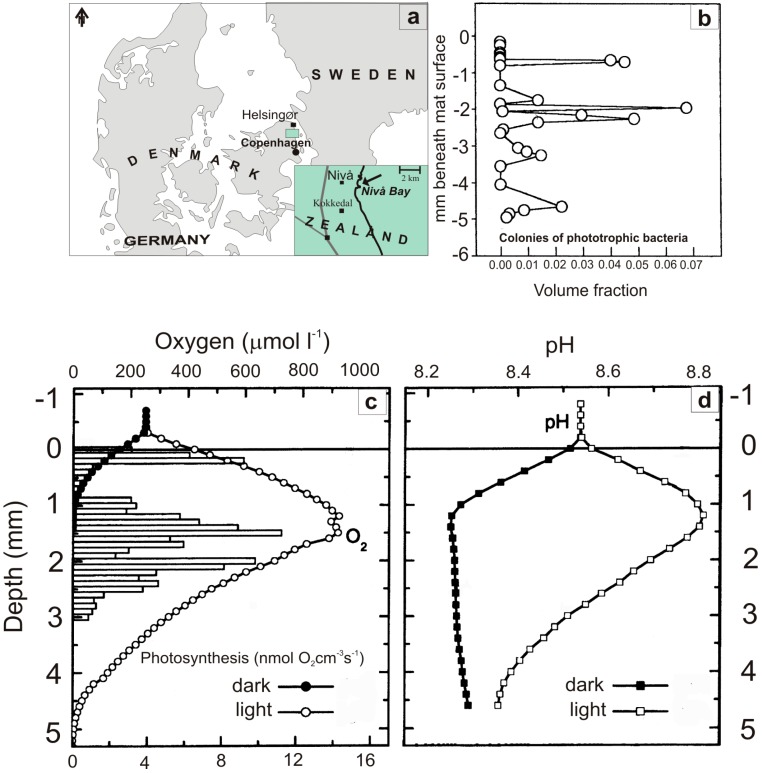
(**a**) Map of Denmark with inset showing Nivå Bay (Øresund), the origin of defaunated sediment samples used for cultivating cyanobacterial mats. (**b**) to (**d**) Microprofiles across a 5 mm thick artificial cyanobacterial mat showing volume fraction of colonies of phototrophic bacteria (**b**), O_2_ concentration (**c**), and pH (**d**) under light and dark conditions (from [49]).

The study is based on sediment samples retrieved in November 1997 from the shallow temperate Nivå Bay (Figure 1a), adjacent to the Øresund, the marine spillway between the North Sea and the Baltic Sea. Macrofauna and most of the meiofauna was inactivated by freezing the sample at ‒20 °C for 20 h and subsequent incubation in sterile filtered seawater [46].

### 2.2. Electron Microscope Analyses

The nano- and microstructures of carbonate morphs in the mat were investigated with a Philips XL-20 scanning electron microscope (Philips, Amsterdam, The Netherlands) (EDS coupled) at 25 kV on hot air-dried mat samples and steel needle hand-picked microsamples sputtered with a layer of platinum 80 Å thick or a layer of carbon 10 to 15 nm thick. For transmission electron microscopy (TEM), mat slices were fixed in 3% glutaraldehyde in phosphate buffer (pH: 7.5), post-fixed in 1% OsO_4_, dehydrated and imbedded in epon. Sections (0.1 µm) were stained in a saturated solution of uranyl acetate in ethanol and viewed with a Zeiss EM900 electron microscope (Carl Zeiss AG, Jena, Germany).

### 2.3. Mineralogical Analyses

The mineralogy of the mat samples was identified by X-ray transmission diffractometry using an Inel instrument with a position-sensitive detector. Carbonate grains from particular mat biozones were hand-picked under a binocular microscope and transferred into Lindemann-brand tubes (0.3 mm in diameter). The diffractograms were recorded using Co K_α1_ radiation at a collection time of 20 h under continuous rotation of the sample. Diffractinel V03/93 software was used to process the obtained data. The content of magnesium in calcite was determined from the d-spacing of the 104 diffraction line [51]. XRD analyses of capillary carbonate micro samples were done with a CGR-INEL diffractometer (Bruker, USA) equipped with a cobalt lamp and a focusing goniometer, and with transmission optics for Debye-Scherrer powder preparations.

### 2.4. Saturation Index of Calcite and Aragonite

The calculated Saturation Index (SI) of calcite and aragonite is the logarithm of the ion activity product of the free calcium [Ca^2+^] and the free carbonate ion [CO_3_^2−^] in solution divided by the solubility constant of the respective minerals at the temperature of measurement:

SI = log([Ca^2+^] × [CO_3_^2−^]/K_calcite,aragonite_) (1)

The logarithm is used so that the SI is negative at undersaturation, zero at saturation, and positive at supersaturation.

### 2.5. Carbon (δ^13^C_carb_) Isotopic Analysis

**Figure 2:**
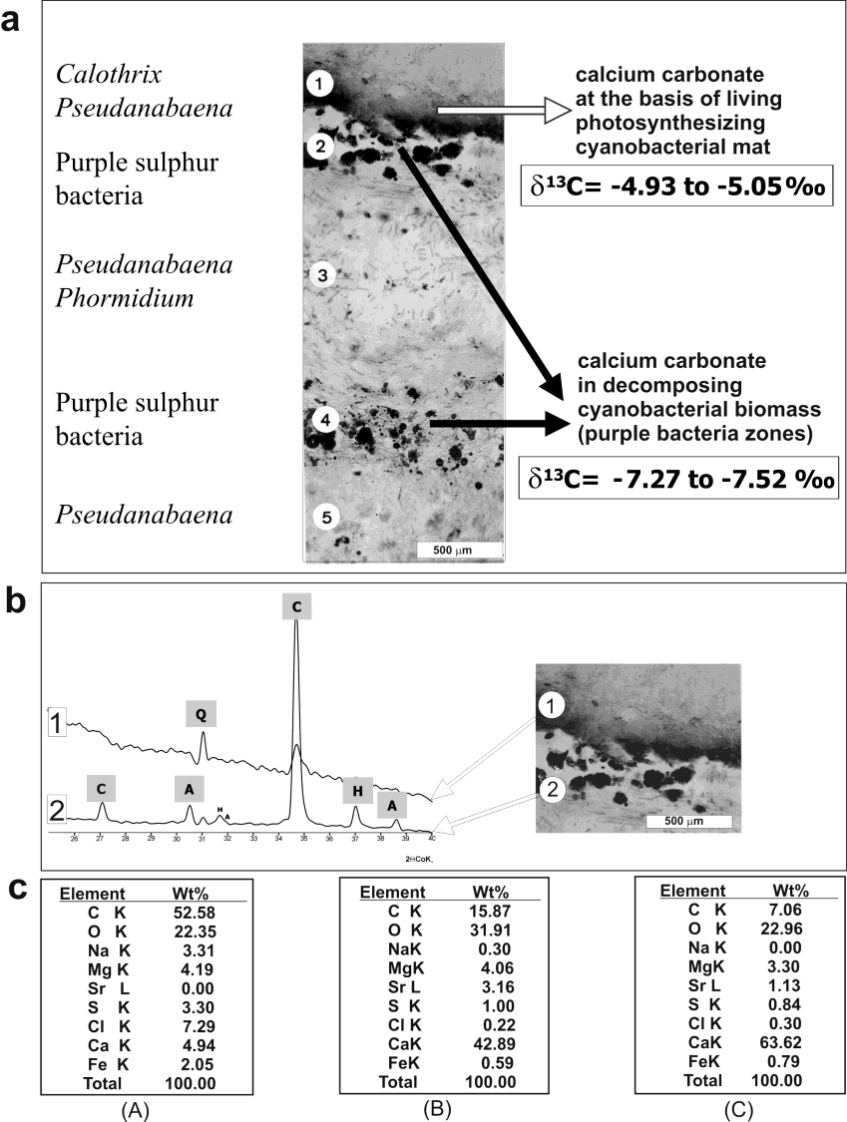
(**a**) Vertical cryomicrotome section of a three-year-old mat showing locations of the CaCO_3_-rich layers (1, 2 and 4) with indicated dominating microbiota and δ^13^C values. (**b**) X-ray diffractograms of mineral components from mat from layers l and 2: C—Mg calcite, A—aragonite, H—halite, Q—quartz (sand grains). The numbers of mat zones correspond to those shown in the diagram in Figure 4. (**c**) SEM-EDS spectra of elemental frequency (wt%) from dried living cyanobacterial biomass from the mat surface layer (A), micrite layer (B), and sparite layer (C).

Stable carbon isotopes of carbonate grains from the artificial mats were analyzed at the Isotope Laboratory of the Institute of Geological Sciences PAS (Warsaw) using a Finnigan MAT Delta Plus mass spectrometer working in dual inlet mode with a universal triple collector. Carbonate precipitates from layers 1, 2 and 4 (see Figure 2a) were selected for carbon isotopic analysis. Two samples were taken from a thin dust-like layer of carbonate at the base of the living surficial cyanobacterial layer (layer 1), and four samples were taken from layers enclosing larger carbonate grains located below the dust-like carbonate layer (layers 2 and 4; Figure 2a). The fragment of the mat selected for isotopic analysis was dried using critical point drying and the carbonate layers were extracted using a scalpel under a dissecting microscope.

It was nearly impossible to remove all organic matter using conventional methods of bleaching (such as treatment with 5% NaClO_4_, 30% H_2_O_2_ and/or roasting under vacuum) because they caused dissolution of the nano-carbonate particles. However, the mat organic matter was tested for its reaction with phosphoric acid to ensure against its influence on the carbon and oxygen isotopic measurement of the carbonate. The selected fragments of the mat were treated with 1N HCl overnight, rinsed with distilled water and dried in an oven at 50 °C. Because of the small amounts of material available, only two runs of the mixture of the calcium carbonate-free mat fragments and Laboratory Standard (WSC) were performed, using the same procedure as for the calcium carbonate. Samples were roasted at a temperature of 420 °C. The weight loss for samples from both layers amounted to 57%. Both samples were then treated with H_3_PO_4_ (density = 1.90 g/cm^3^) and heated to 220 °C with the addition of H_2_O_2_ and CrO_3_ solutions. Total calcium carbonate (calcite and aragonite) was converted to CO_2_ by treating mat fragments 2‒4 mm in size with orthophosphoric acid (d = 1.90 g/cm^−3^) at 25 °C for 1 hour in vacuum, and purified by cryogenic distillation. The isotopic ratios were corrected by a factor of 1.01025, *i.e.*, the isotopic fractionation during phosphoric acid reaction, which is nearly the same for calcite and aragonite [52]. Isotopic ratios are expressed in δ notation relative to the VPDB standard. The precision of the results of carbon isotopic composition of WSC are within laboratory precision: 0.05% and 0.1%, respectively.

Additionally, the stable carbon isotopes from a series of alternating micrite and sparite layers of the subfossil stromatolites from the quasi-marine crater lake Mototoi (Satonda Island, Indonesia) were measured in the BP International Ltd., Sundbury Research Center, Sunbury-on-Thames (UK) with laser ablation sampler for stable isotope extraction (LASSIE). To ablate the samples of areas less than 50 μm directly from a thin section LASSIE utilized a high power (~65 W at full power) Nd:YAG laser which generated beam of infrared light with a wavelength of 1064 nm. The CO_2_ gas produced (with minor CO, O_2_ and H_2_O) was passed into a vacuum line, purified in a cold trap and passed into a Finnigan MAT 251 mass spectrometer (equipped with an 80-*μ*L micro-inlet for analysis (for procedure see [53]).

## 3. Previous Studies

The formation, biology and biogeochemistry of artificial microbial mats have previously been studied in detail [46,47,48,54]; thus, we present only an overview here. A more extensive review along with comments on the calcification potential of the mats was presented elsewhere [50].

### 3.1. Structure and Development

Three to four weeks after freezing inactivation, the growth of an extensive cyanobacterial mat on top of the sediment was observed. After four months of incubation at lab temperature and with a 10 h dark/14 h light illumination cycle, a laminated, cohesive, yellow-brownish matrix of cells and exopolymers had formed. It was composed of various phototrophs (mainly different filamentous cyanobacteria and colonial purple bacteria) that predominated in different layers of the mat (Figure 1D). Furthermore, the first signs of ongoing carbonate precipitation appeared in the upper 0.5 mm of the matrix [47]. Over several years, the stratified microbial mat developed further in complexity, showing a growth rate of 2‒3 mm a^−1^, a carbonate accumulation rate of ~0.1 mmol cm^−2^ a^−l^, and an organic carbon accumulation rate of ~0.9 mmol cm^−2^ a^−l^ [54]. Most of the net growth was due to accumulation of exopolymers and empty cyanobacterial sheath material, which appeared to be largely resistant to degradation. Carbonate precipitation on and in the vicinity of cyanobacterial sheaths was also observed. The total photopigment content of the mat approached an asymptotic level of ~150 μg chlorophyll *α* cm^−2^ and ~25 μg bacteriochlorophyll *a* cm^−2^. The 3‒5-mm-thick zone of active photosynthetic microbes moved upwards as the mat thickness increased [46,50]. Within 1‒1.5 year (2001/2002), the mat developed conspicuous gelatinous pinnacles on its surface [50]. This massive excretion of exopolymers may indicate unbalanced growth due to nutrient limitations in the upper photosynthetic layer [55].

In summary, the artificial mats exhibit prominent characteristics found in extant natural mats and often interpreted as preserved in fossil material (e.g., [55,56]): (i) a pronounced microfabric of cyanobacterial lamina, where layers with vertical filament orientation are separated from layers with pronounced horizontal filament orientation, (ii) an abundance of empty cyanobacterial sheaths that are not mineralized and, together with exopolymers, constitute the bulk of the mass constituting the mats, and (iii) the presence of distinct layers of carbonate precipitates associated with photosynthetic zones and zones of mainly heterotrophic activity.

### 3.2. Microenvironmental Characteristics and Carbon Cycling

In addition to a strong structural similarity to natural microbial mats, the physico-chemical microenvironment (Figure 1B,C) and the cycling of carbon, oxygen, sulfur, and other elements in the artificial mat were similar to observations made in natural marine microbial mats (for an extensive review see [55]. In darkness, the diffusion boundary layer limited the supply of O_2_ for respiratory processes in the mat, and O_2_ penetrated only 0‒1 mm into the mat [48,49]. In light, this diffusion limitation was alleviated by intense internal O_2_ production by the cyanobacteria, and O_2_ penetrated to depths of 3‒5 mm below the mat surface (Figure 1B). The photic zone of oxygenic photosynthesis was limited to the uppermost 3 mm due to intense attenuation of visible light in the cyanobacterial layers, while near-infrared light penetrated into deeper mat layers with purple bacteria [49]. Below the oxygenated zone (but also within the photic zone), sulfate reduction produced sulfide, which was re-oxidized at the oxic-anoxic interface by chemotrophic bacteria and purple photosynthetic bacteria. Thus, O_2_ and sulfur cycling were closely coupled in the mats.

In accordance with the slow accumulation of organic carbon (~10% of net carbon fixation), net uptake in light and net production in darkness of inorganic carbon was almost identical, showing efficient recycling of carbon, sulfur and other elements within the mat [45]. However, net O_2_ fluxes in light and darkness did not balance out, and were lower than the inorganic carbon fluxes, as O_2_ was also used to reoxidize reduced carbon and sulfur pools in darkness and cyanobacterial storage products accumulated in the light [48]. In addition, a stimulation of O_2_ respiration in illuminated mats was found; this was probably due to the excretion of photosynthates by cyanobacteria stimulating heterotrophic activity in the immediate vicinity of the phototrophs [49].

## 4. Mat Microbial Zones and Associated CaCO_3_ Morphs

**Figure 3:**
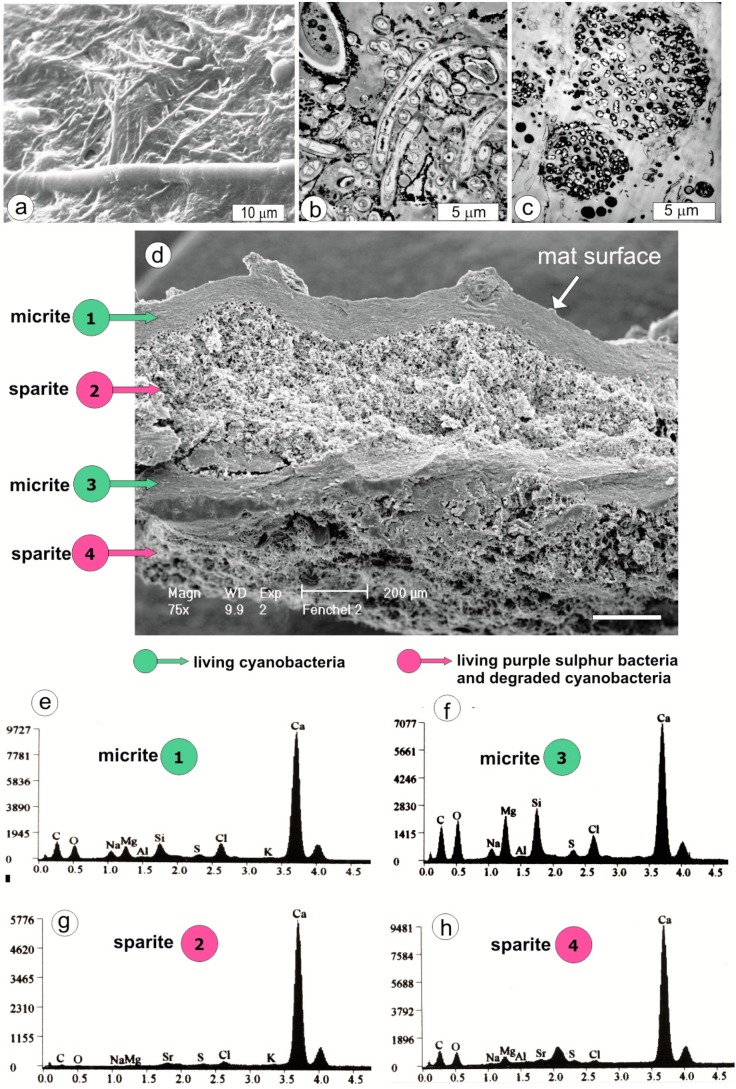
(**a**) SEM image of the artificial mat surface (hot air-dried shrunken specimen) showing thinner filaments of *Pseudanabaena* and thicker filaments of *Calothrix*. (**b**) TEM image of *Pseudanabaena* and *Calothrix* filaments from about 0.3 mm below the mat surface. Note the large volume of extracellular polymers (EPS) excreted by the cyanobacterial filaments, which are incidentally associated with much smaller heterotrophic bacteria, and more rarely Mg calcite nanograins (opaque matter) precipitated in larger accumulations particularly on *Calothrix* filament (upper left corner). (**c**) TEM image of colonies of purple sulfur bacteria (possibly *Thiocapsa*) characteristic of the mat layers 2, 4, 6 and 8 (see diagram in Figure 4). (**d**) Vertical section of hot air-dried fragment of the artificial mat showing shrunken and weakly mineralized living cyanobacterial layers (micrite) alternating with strongly mineralized layers (sparite) composed of degraded cyanobacteria (mostly empty sheaths) and purple sulfur bacteria. (**e**, **f**) SEM-EDS spectra to show the negligible presence of Mg silicate in Mg calcite from the surficial cyanobacterial layer (micrite 1) and much higher in the Mg calcite from deeper located cyanobacterial layer (micrite 3).

The present study is based on observations made on cryomicrotome sections of approximately three-year-old mats (Figure 2), the structure of which is outlined in Figure 3. The figure illustrates schematically the biotic structure and corresponding mineralogical composition of the mats. Scanning electron microscope images of the CaCO_3_ precipitates occurring in particular microbial zones showed that the morphologies and modes of distribution of carbonate grains were not random but tied to well-defined zones labeled from 1 to 4 in Figure 3. Thus, in the ca. 7 mm-thick mat, several carbonate layers could be easily distinguished.

**Figure 4:**
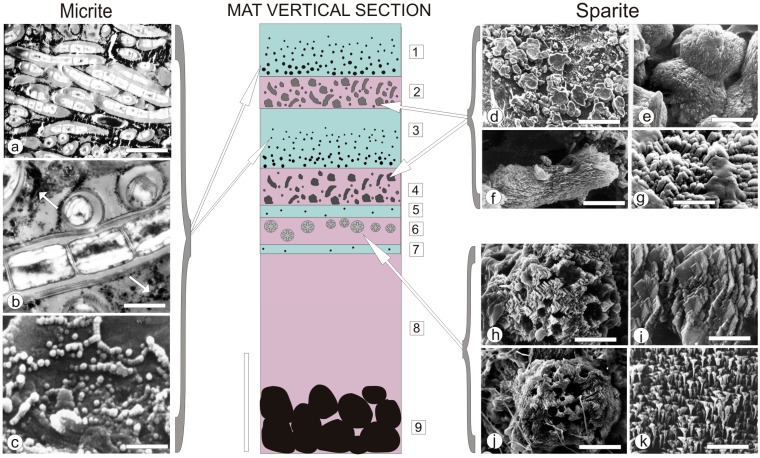
A schematic presentation of the artificial mat zonation (in vertical section), with examples of most characteristic calcium carbonate morphs precipitated in micritic (left column) and sparitic (right column) layers (modified after [50]). For detailed explanation see text. Scale bars: (a) 5 μm, (b) 1 μm, (c) 1 μm, (d) 100 μm, (e) 10 μm, (f) 10 μm, (g) 10 μm, (h) 10 μm ,(i) 3 μm, (j) 20 μm, (k) 2 μm.

From a sedimentological point of view, two types of calcium carbonate precipitates were identified in the studied mats, which can be classified as precursor grains for **micrite** and **sparite** layers (Figure 3 and Figure 4):

(i) **Micrite:** <1 µm-sized globular grains of Mg calcite (occasionally with an admixture of nanograins of magnesium silicate) precipitated in the mucilage of the living cyanobacterial layers, particularly dense at their basal parts.
(ii) **Sparite:** a variety of >1 µm-sized mixed Mg calcite/aragonite particles (anhedral and subhedral grains, hemispheres, dumbbell- and peanut-like bodies, and subglobular aggregates of dagger-like and rhombic, platy crystals) precipitated in the zones of cyanobacterial necromass colonized predominantly by phototrophic purple bacteria with varying admixtures of green sulfur bacteria and heterotrophic bacteria.

As shown schematically in Figure 4, the mat consists of nine, more or less distinct layers, eight of them characterized by a community of specific microbiota, and some by an assortment of calcium carbonate grains with often well-defined morphology (morphs).

Layers 1 and 3, which are composed of living filamentous cyanobacteria, contained submicrometer-sized carbonate grains corresponding by size to the definition of **micrite**. The surface layer 1 was composed largely of mucilage embedding trichomous cyanobacteria identified as *Calothrix* and *Pseudanabaena* mixed with heterotrophic bacteria. At the base of this *living* mucilage-rich cyanobacterial layer, an almost continuous layer of spherical submicrometer-sized CaCO_3_ grains occurred (Figure 2a–c). These 50‒750 nm-sized particles were distributed both on the slime sheaths of living cyanobacterial filaments and within the extracellular polymers (EPS) tightly filling the space between the filaments. Most particles occurred as separate bodies, but loosely connected chains and clusters were also observed (Figure 4c). X-ray diffractometry showed that they were composed of high-Mg calcite (15‒17 mole% MgCO_3_) (Figure 2b) and, as EDS analyses showed, with a small admixture of nanograins of magnesium silicate. In layer 3, *Calothrix* was lacking and the cyanobacterial community was predominated by *Pseudanabaena* and *Phormidium* filaments. The density of the ultra-small high-Mg calcite grains was much lower in this layer as compared with layer 1. The carbonate nanograins occurring in the very thin layers 5 and 7, both composed of living *Pseudanabaena,* were too rare for XRD analysis.

In contrast to the fine-grained (**micritic**) calcium carbonate precipitates occurring in the living filamentous cyanobacterial layers, the layers of variously degraded cyanobacterial necromass were dominated by purple sulfur bacteria and heterotrophic bacteria (layers 2, 4 and 6). The coarse-grained (**sparite**) calcium carbonate precipitated in these layers included larger (2‒4 µm) anhedral and subhedral grains of Mg calcite with an admixture of aragonite, some showing crystal faces (Figure 4h,i). Button-like, 20‒10 µm large Mg calcite bodies were common and these often coalesced into plate-like groups (Figure 4d). Groups of Mg calcite/aragonite peanut-shaped (Figure 4f) and dumbbell-like morphs (Figure 4e), 20‒50 µm in size, were also occasionally noticed. All these grains were usually found entangled in remnants of empty cyanobacterial sheaths. Purple sulfur bacteria were also dominant in Layer 6, which was rich in coarse-grained (**sparite**) carbonate particles. Here the main CaCO_3_ morphs were 50‒120 µm large globular and subglobular grains composed of dagger-like crystals (Figure 4j,k) and some aggregates of platy, rhomb-like crystals (Figure 4h,j). The grains and aggregates were mostly composed of aragonite with a small admixture of high-Mg calcite. They were loosely distributed in felt-like remains of sheaths of filamentous cyanobacteria.

The deepest, 2 mm-thick, layer 8 was composed of discrete colonies of purple sulfur bacteria irregularly distributed in the mucilaginous matter. No carbonate particles were observed in this zone similarly or in layer 9 corresponding to a layer of blackish sulfidic fine quartz sand substratum (Figure 4, diagram) where activity of sulfate reducing bacteria was recorded.

## 5. Carbonate System in the Artificial Mat

The calcite, aragonite, and dolomite saturation values for the pore waters of the studied mats showed very high supersaturation values in the uppermost 2‒3 mm of the mat (Table 1). These high values were related to the much higher pH and carbonate alkalinity levels in the mats and an increased concentration of Ca^2+^ and Mg^2+^ liberated presumably from the decaying, Ca- and Mg-enriched cyanobacterial sheaths and extracellular polymeric substances (EPS).

**Table 1:** Hydrochemistry, carbonate system, and saturation indices (SI) calculated for a seawater sample from Nivå Bay (used in the experiment), and particular depth zones in the artificial mat cultured over three years (in part from [50], recalculated).

**Nivå Bay**: Calculated for S = 22‰ and T = 15°C; Ca: 6.3; Mg: 33.3; K: 6.3; Na: 295.4; SO_4_: 17.6 (all mmol/L)							
Alk_c_		8.49 meq/L		SI _Calcite_		0.67	
pH		7.80		SI _Aragonite_		0.52	
pPCO_2_		2.35		SI _Dolomite_		2.10	
δ^13^C -3.09							
**Artificial mat:** Calculated for S = 21‰ and T = 20°C; Ca: 6.0; Mg: 31.8; K: 5.16; Na: 270.9; SO_4_: 15.6 (all mmol/L)							
			Light		Dark		
	above mat		1 mm depth	2 mm depth	1 mm depth		2 mm depth
pH	8.54		8.82	8.73	8.25		8.26
Alk_c_	8.54		10.10	6.58	10.10		6.50
SI _Calcite_	1.48		1.73	1.48	1.18		1.00
SI _Aragonite_	1.33		1.58	1.33	1.03		8.85
SI _Dolomite_	3.79		4.29	3.79	3.11		2.76
pPCO_2_	3.04		3.35	3.43	2.72		2.92

S = salinity; T = temperature; Alk_c_ = carbonate alkalinity (meq/L); pPCO_2_ = negative log of partial pressure of CO_2_. Saturation Indices (SI) were calculated according to the formula: SI = log ([Ca^2+^] × [CO_3_^2−^]/K) with the software PHREEQE [56].

## 6. Discussion

The distribution of different carbonate precipitates in the studied mats indicates that two processes were involved in their formation:

One process involved precipitation of ultra-small (<1 μm) high-Mg calcite grains associated with living cyanobacterial layers. The nucleation in such layers was most probably a spontaneous process resulting from the high pH values induced by photo assimilatory uptake of CO_2_ and/or HCO_3_^−^ by cyanobacteria, causing a significant increase in the carbonate ion and thus in the CaCO_3_ saturation level (Table 1). In many places, the precipitated nanograins formed subglobular or irregular aggregates, in which they lost their individuality and merged into almost structure less Mg calcite blobs.

The second process of CaCO_3_ precipitation took place in zones occupied predominantly by purple bacteria and heterotrophic bacteria, with the latter degrading the cyanobacterial necromass. This probably less viscous precipitation environment favored the formation of larger carbonate particles of mixed Mg calcite/aragonite mineralogy. As in the living cyanobacterial layers, the nanometer-sized precursor particles were transformed much faster into large superstructured grains composed of ordered nanograins and nanocrystals (Figure 4g). It is known that such small and initially even amorphous CaCO_3_ particles (ACC) are unstable and rapidly transform into macrocrystals [57,58,59,60,61,62].

The presence of two different microenvironments of calcium carbonate production within the mats is supported by the results of stable carbon isotope analyses of the CaCO_3_ layers (Figure 2). They showed δ^13^C values about 1‰ to 2‰ heavier for carbonate layers generated in the living cyanobacterial zones (due to photosynthetic uptake of lighter carbon) as compared with layers generated in zones of decaying cyanobacterial biomass (due to input of lighter respiratory carbon).

The saturation indices measured in the uppermost 2 mm of the studied mats showed very high supersaturation (Table 1). It has been shown [3,4,63,64,65,66,67,68,69,70,71] that high calcium carbonate saturation levels in the water above the cyanobacterial mats (SI over 0.8)—see [4,65,72] are most probably the crucial factor controlling their calcification potential.

In Nivå Bay (Øresund, Denmark), from which the environmental samples originated, no carbonate precipitation was observed. The appearance of a spectrum of calcium carbonate morphs in the cultured mats was thus rather unexpected. Calcification is a function of a supersaturation. Supersaturation depends in turn on the concentrations of free Ca and CO_3_ ions. The Ca concentration in Nivå Bay was, at a salinity of 22 ppt, lower (by a factor of 22/35) than in average seawater of 35 ppt. On the other hand, the alkalinity of >8 meq L^−1^ in Nivå Bay was much higher than in average seawater (which would be less than 2 meq L^−1^, considering the low salinity). This is most probably a consequence of sulfate reduction in the sediments of the shallow bay. High alkalinity, however, is not a direct measurement of carbonate concentration, but the sum of the negative charges of the bicarbonate and carbonate ions. The partitioning between the two ions is governed by CO_2_ pressure, which in turn determines pH. In the present paper we have not considered the potential contribution of nitrate, ammonium and orthophosphate in alkalinity measurements. In a closed system the mats were cultured, any excessive influx of the anions was barely possible for a longer time. Thus, measuring pH, temperature, and alkalinity and calculating the other main ion concentrations, among them calcium, from the measured salinity enables the calculation of the SI. High alkalinity *per se* does not lead to a high SI because the pH is—for average surface seawater—relatively low, possibly because of ongoing respiration in the sediments (Table 1). Even average surface seawater, in spite of its low PCO_2_ (and therefore high pH) and high Ca concentration, maintains an SI below 0.8 because of its relatively low alkalinity. Thus, carbonate precipitation can normally only be triggered if seawater is concentrated by substantial evaporation such as that occurring in highly evaporative (hypersaline) lagoons or intertidal flats and embayments [8,12,13,22,27]. This is a paradox when considering the abundance of carbonate deposits produced apparently by *in vivo* and/or early post-mortem calcified benthic cyanobacterial mats in ancient seas (e.g., stromatolites, thrombolites and some fine-grained and peloidal limestones) [35,73,74,75,76].

The studied artificial mats provide insight as to why morphologically identifiable remains of mat-forming microorganisms are rarely preserved in carbonate stromatolites. It seems that the morphology of the mat-forming microbiota has a chance to be preserved only if the carbonate precipitating process is intense and fast enough to cover or fill in the mucilage (EPS), creating mineral coatings on cells or groups of cells. Apparently such mineral covers must be formed before bacteriolysis destroys the original (*i.e.*, living) cell configuration. Identification of the primary morphology of cyanobacteria in calcified mats is much easier in case of filamentous forms and difficult, or often impossible, in the case of coccoid cells that are usually much smaller. In these cases even slight re-crystallization of the primary fine-grained CaCO_3_ is a destructive factor (e.g., [23]). We conclude that the fossilization potential of cyanobacteria in the studied mats is very low, as they are degraded to an almost unidentifiable mass of organic matter.

The majority of the primary CaCO_3_ morphs in the studied mats seem to be transient in terms of their preservational potential. From the known microstructural and textural characteristics of ancient carbonate stromatolites and thrombolites studied thus far (for review: [35,73,74,77]), no carbonate morphs similar to those from the artificial mats have been observed. It can be deduced that the primary nanometer-sized morphs as those observed in the studied living cyanobacterial layers (Figure 4b,c) are rather quickly diagenetically transformed (super-structured) in natural settings to micrometer-sized fine-grained (microcrystalline) Mg calcite (**micrite**), whereas the larger aragonite/Mg calcite morphs precipitated in the purple bacterial zones recrystallized into a more or less uniform mosaic of Mg or low-Mg calcite crystallites (**sparite**).

**Figure 5:**
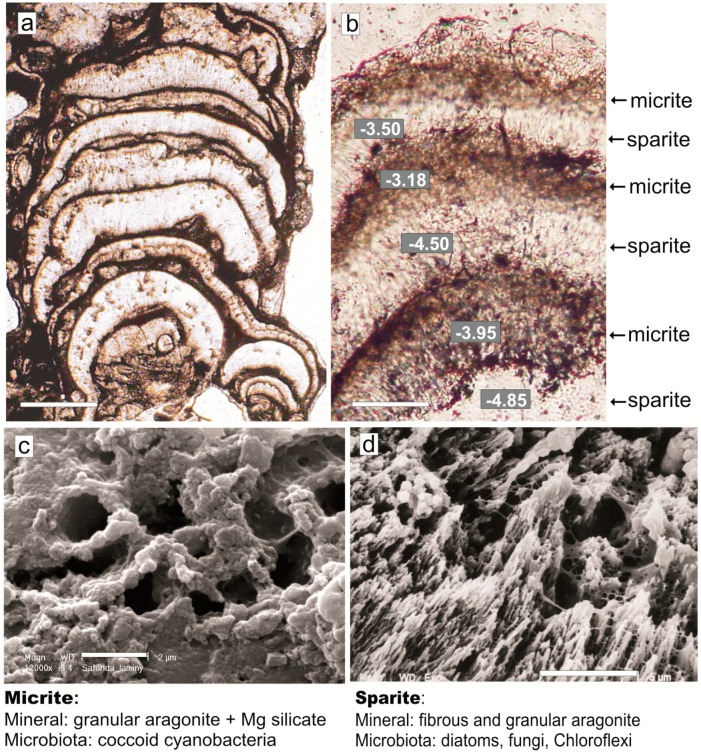
Subfossil microstromatolite from the quasi-marine Satonda Crater Lake (Central Indonesia) interpreted as the product of a multilayered coccoid cyanobacterial mat analogous to the studied artificial cyanobacterial mat. (**a**) Optical micrograph of vertical petrographic thin section of the Satonda stromatolite showing well-expressed alternation of micritic and sparitic layers. (**b**) Magnification of a series of alternating micrite and sparite layers with indicated stable carbon isotope (δ^13^C *vs*. PDB) signatures. (**c**) and (**d**) SEM images of vertical sections of polished and with 5% formic acid etched micritic (c) and sparitic (d) layers; note the well-preserved, due to *in vivo* mineralization, pattern of the common mucilage sheaths (glycocalyx) in the micritic layer, and the almost totally degraded remains of mucilage sheaths in the sparitic layer. Scale bars: (a) 500 μm, (b) 200 μm, (c) 2 μm, (d) 5 μm.

**Figure 6:**
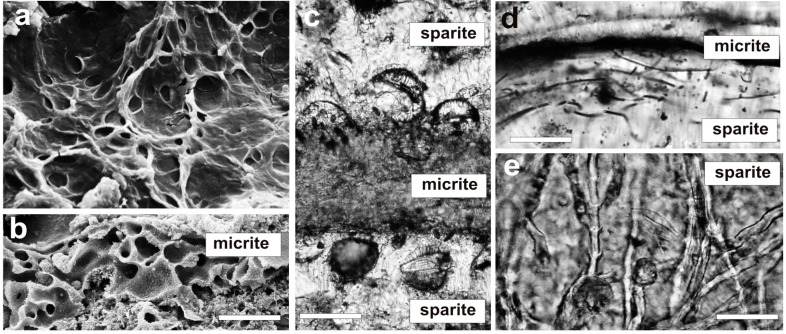
Microbiota associated with calcium carbonate layers in microstromatolites from the quasi-marine Satonda Crater Lake (Central Indonesia). (**a**) SEM image (top view) of a coccoid pleurocapsalean cyanobacterial mat growing today in the lake. (**b**) SEM image of vertical mat section showing mucilage sheaths (glycocalyx) of coccoid pseudocapsalean cyanobacteria permineralized *in vivo* with aragonite nanograins (micrite). (**c**–**e**) Micrographs of thin sections showing examples of remains of microbiota associated with aragonite layers (sparite) precipitated in decomposing biomass of coccoid cyanobacteria: diatoms (c), *Chloroflexus*-like bacteria (d) and aquatic fungi. Scale bars: (a) 20 μm, (b) 20 μm, (c) 10 μm, (d) 10 μm, (e) 20 μm.

An instructive example of such diagenetic transformation of the primary calcium carbonate morphs in **micrite** and **sparite** layers are subfossil (micro) stromatolites from the quasi-marine Satonda Crater Lake (Central Indonesia) formed by colonial coccoid (pleurocapsalean) cyanobacteria [5,11,78,79]. The lamination in these stromatolites is extremely well-expressed, whereby the micritic laminae are as a rule thinner than the sparitic ones (Figure 5a,b). The micritic laminae are composed of aragonite nanograins, with some admixture of Mg silicate nanograins, both apparently precipitated in the common mucilage sheaths (glycocalyx). A few diatoms were found in these laminae between colonies of coccoid cyanobacteria. Similarly as in the case of the studied artificial mats, the microbiota from the micritic laminae (Figure 5c) indicate an aerobic character of the microenvironment sustaining the origin of the aragonite nanograins. The sparitic laminae in turn consisted of vertically well-ordered aragonite fibres which, as indicated by the presence of highly degraded remains of sheaths of coccoid cyanobacteria (Figure 5d), precipitated in a decaying cyanobacterial necromass. The remains of filaments of Chloroflexi-like phototrophic bacteria, partially desilicified diatom frustules and saprophytic fungi abundant in the sparitic laminae suggest, similarly as in the artificial mats, a dysoxic or anoxic microenvironment conditions for these laminae (Figure 6). It is known that the well-laminated Satonda Crater Lake stromatolites developed in a quasi-marine environment (Table 2) with increased alkalinity that is characterized by alternating dry and wet seasons [5,11,78] controlling not only mat growth but possibly also enforcing two basically different calcifications processes observed in the mats: (i) the *in vivo* precipitation of **micritic** aragonite in the living cyanobacterial layer due to high CaCO_3_ saturation in the mat ambience during dry season, and (ii) the post-mortem precipitation of **sparitic** aragonite in the decaying layers of coccoid cyanobacteria growing uncalcified or weakly calcified during the wet season when CaCO_3_ saturation in the mat ambience was lower. Stable carbon isotope (δ^13^C) signatures from neighboring individual laminae of the Satonda stromatolites, obtained with laser ablation sampler for stable isotope extraction (LASSIE) (Figure 5b), show values consistent with those obtained from the studied cultured mats (Figure 2). In both cases the values from the micritic laminae were almost always 0.5‰ to almost 2‰ heavier than those from the underlying sparitic laminae. This suggests that the results of δ^13^C measurements made on bulk samples of fossil stromatolites may not exactly reflect the stable carbon isotopic signals in the water of the mats ambience (for discussion see e.g., [63,64,80,81,82]).

**Table 2:** Basic chemical and isotopic data of Satonda Crater Lake and seawater from the shore of Satonda Island *.

	Satonda Crater Lake		Seawater (Satonda shore)
	above chemocline	below chemocline **	mean values
pH	8.42	7.31–6.87	8.20
alkal_t_	3.5 meq/kg	5.6–50 meq/kg	2.1 meq/kg
pCO_2_	372 ppmv	10,000–240,000 ppmv	309 ppmv
Na	425 meq/kg	478–560 meq/kg	460 meq/kg
Mg	86 meq/kg	96–110 meq/kg	102 meq/kg
Ca	10 meq/kg	12–13 meq/kg	21 meq/kg
Mg/Ca	7.4	7.8 ***	4.8
SI_Cc_	0.84	0.09–0.61	0.73
SI_Ara_	0.70	0.54–0.61	0.59
δ^13^C	−8.4 (10 m)	−11.4 (40 m); −21.4 (60 m)	-
δ^18^O	3.05 (10 m)	2.99 (40 m); 2.53 (60 m)	-

* For detailed information on the lake chemistry see [78]; ** Unless other indicated, values for water samples taken from depths of 30 m and 60 m respectively; *** Mean value. Saturation Indices (SI) were calculated according to the formula: SI = log ([Ca^2+^] × [CO_3_^2−^]/K) with the software PHREEQE [56].

Concerning the mineralogical stability of the CaCO_3_ polymorphs both from the studied artificial mats and those from the subfossil Satonda Crater Lake stromatolites, it can be supposed, by comparison with diagenetic processes recognized in other calcium carbonate sediments [83] that, with time, they will transform into more stable calcium carbonate polymorphs: the high Mg calcite of the micritic laminae into low Mg calcite and the aragonite of the micritic and sparitic laminae into calcite.

The studied artificial mats were cultured under constant environmental lab conditions in terms of temperature, illumination, basic hydrochemistry and absence of macrobiota. Therefore, their biological and mineralogical zonation cannot be explained with the mode of origin suggested for the Satonda Crater Lake stromatolites and for many fossil calcareous stromatolites [76,84,85,86,87] linking alternation of fine-grained (**micritic**) and coarse-grained (**sparitic**) layers to periodic environmental changes in the mat ambience. Much more laboratory and field studies are still needed to fully clarify which factor(s) are responsible for the origin of the micrite/sparite lamination in multilayered microbial mats like those presented herewith.

Our current understanding of the mechanisms involved in the microbial zonation (and associated biomineralization) occurring in the studied multilayered mat is as follows: The growing surficial cyanobacterial layer is from time to time interrupted by a community of photosynthetic purple bacteria that rapidly create their own microenvironment, while the associated heterotrophic bacteria start to decompose the dead cyanobacterial biomass. The purple bacteria and associated heterotrophic bacteria divide the cyanobacterial layer in two (or even more) layers that still continue to grow. Weakly calcified, multilayered microbial mats described from modern natural settings may show similar dynamics [16,17,28,88,89]. As discussed above, the purple bacterial layers create conditions favoring precipitation of larger aragonite/Mg calcite morphs (for detailed discussion [90,91]. Photosynthetic purple bacteria can use H_2_, H_2_S, S_o_, or certain organic compounds as electron donors. Depending on the character of the donor, the amount of CO_2_ released during photosynthesis may be higher than that assimilated. Due to uptake of the CO_2_ excess from the purple bacteria zones by the photo-assimilating cyanobacteria and diatoms, increased pH and alkalinity (carbonate ion abundance) is generated in the upper 2‒3 mm of the mat. Altogether, this causes high carbonate supersaturation resulting in precipitation of a variety of CaCO_3_ particles, some with characteristic morphologies (hemispheres, dumbbells, and peanut-shaped morphs). The much larger dimensions of these particles, as compared with the sub-micrometer grains from the living cyanobacterial layers, probably result from higher nucleation kinetics in the less viscous medium as compared to the viscous EPS of the living cyanobacterial layers. High-nucleation kinetics media are favorable for faster transformation of the nanoprecursor grains into super-structured morphs built of ordered monocrystals (e.g., [59,60,61,62,92,93,94,95]). As noted by previous authors [96,97,98], the appearance of admixture of aragonite in the high-Mg calcite forming the bulk of the grains in the purple bacterial zones is also in line with the less viscous character of the precipitation microenvironment. However, in other experimental studies the main control of the great variety of CaCO_3_ morphs precipitated from pure inorganic calcium carbonate supersaturated solutions is simply exerted by pH changes [94,95,99,100].

The CaCO_3_ precipitates produced by the studied cyanobacterial mats are similar to CaCO_3_ morphs obtained not only in experiments with bacterial cultures and bacterially decomposed cyanobacterial biomass kept in artificial Ca-rich media, but also to grains precipitated from sterile, calcium carbonate supersaturated solutions (for review see: [60]). This seems to contradict the metabolically active role of heterotrophic bacteria in the precipitation of CaCO_3_ within cyanobacterial mats claimed by some researchers.

**Figure 7:**
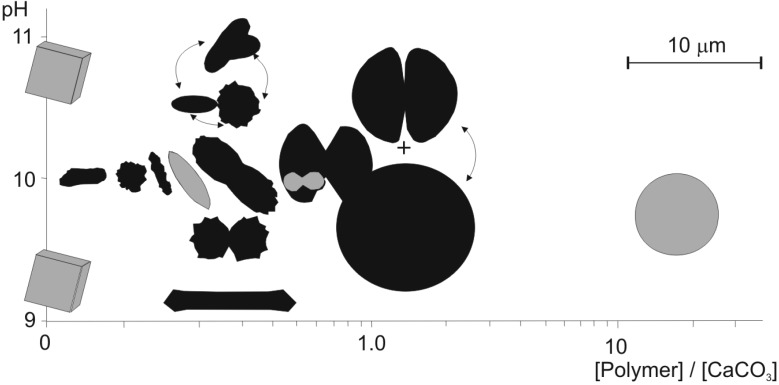
Diagram showing a variety of CaCO_3_ morphs precipitated under controlled conditions from a solution of different pH and [polymer]/[CaCO_3_] ratio for which the unit for both concentrations is g L^−1^. Arrows indicate morphologies observed simultaneously. Morphs obtained from the same system are drawn in gray and those from other experiments are drawn in black (from [58], slightly modified).

Bacterial mediation has been suggested in studies on CaCO_3_ precipitates obtained from cyanobacterial mats decaying in calcium-rich solutions [96,97,101,102]. The morphologies of the CaCO_3_ particles from these experiments are strikingly similar, if not identical, to the spectrum of morphs generated in our mats. Spheres, hemispheres, dumbbells, and peanut-shaped particles have also been produced in cultures of heterotrophic bacteria growing in CaCO_3_-supersaturated solutions with and without addition of Mg (e.g., [103,104,105,106,107,108,109,110]). However, none of these studies provided unequivocal evidence for a metabolically active role of bacteria in the precipitation of the morphs. Moreover, a whole array of CaCO_3_ morphs has been obtained from inorganic solutions after the controlled addition of various polymers [57,58,111,112,113,114,115,116,117,118,119,120,121] It seems therefore that the role of heterotrophic bacteria in the process of CaCO_3_ precipitation is mainly to supply the calcium-carbonate-oversaturated medium with polymers effective in crystallization control of the final morphologies (morphs) of the CaCO_3_ particles (Figure 7).

The bacteria—or their remains—could, to some extent, also serve as nucleation centers for the crystallization of the CaCO_3_ morphs [122,123,124,125,126,127,128]. However, the importance of heterotrophic bacteria degrading the dead cyanobacterial biomass or, as some studies suggest, infesting and killing the cyanobacterial community [129], concerns probably mainly their lytic action upon the cyanobacterial biomass [130] releasing various classes of polymers and liberating *in vivo* EPS-bound (complexed) Ca^2+^ and Mg^2+^ cations to the precipitation medium and significantly increasing its CaCO_3_ saturation level (e.g., [22,60,131,132,133,134,135]. An enrichment of Ca and Mg in the living non-mineralized cyanobacterial layer of the studied mat measured with SEM-EDS (4.94 and 4.19 wt% respectively—see Figure 2c, panel A) and on dried EPS free CaCO_3_ grains with sparse trichomes support these suggestions. The SEM-EDS elemental analyses of the calcium carbonate grains from the micritic and sparitic layers showed the highest Ca content in the sparitic layer (Figure 2c, panels B and C). Due to advanced decomposition of the cyanobacterial necromass in this layer, the input of desorpted calcium cations to the precipitation fluid was apparently most intense. Thus, as newly evaluated [136], Ca^2+^ concentration in the CaCO_3_ precipitating fluids in microbial mats maybe a critical process contributing to calcium carbonate oversaturation in the calcifying microbial communities as an alternative to sulfate reduction favored by many researchers.

## 7. Conclusions

Our study showed that precipitation of CaCO_3_ morphs in cyanobacterial mats can occur ***simultaneously*** in metabolically active cyanobacterial layers as well as in biomass of degrading mat layers. Morphs precipitating from living layers are represented by ultra-small (<1 μm) high-Mg calcite grains (***micrite layers***), whereas those precipitated in layers with dead (biodegraded) cyanobacteria settled by purple and green sulfur bacteria in association with heterotrophic bacteria are represented by a great variety of >1 μm-sized particles often of mixed high-Mg calcite/aragonite composition (***sparite layers***).

The alternation of *in vivo* and post-mortem generated calcareous layers clearly visible in the studied mats may help to explain the alternation of fine- and coarse-grained (**micritic** and **sparitic**) laminae observed in many modern, subfossil and fossil calcareous cyanobacterial microbialites, as the result of a probably similar multilayered biological organization of the precursor mats.

The process of CaCO_3_ precipitation in the artificial mats is enhanced and controlled by seawater with higher calcium carbonate saturation levels comparing with average seawater. This may suggest that the past epicontinental seas sustaining often *in vivo* calcifying cyanobacterial microbialites (stromatolites), and cyanobacteria-associated fine-grained and peloidal limestones were characterized by significantly higher calcium carbonate saturation level than present-day seawater.

The CaCO_3_ morphs identified in the artificial mat are comparable with CaCO_3_ morphs precipitated not only in other natural and artificial microbial mats, but also with morphs originating in laboratory experiments in calcium carbonate oversaturated solutions both with and without organic additives. Notably, no metabolically controlled activity of heterotrophic bacteria in the formation of CaCO_3_ morphs was observed in the studied mats.
